# Deoxynivalenol Exposure in Norway, Risk Assessments for Different Human Age Groups

**DOI:** 10.3390/toxins9020046

**Published:** 2017-02-04

**Authors:** Leif Sundheim, Inger Therese Lillegaard, Christiane Kruse Fæste, Anne-Lise Brantsæter, Guro Brodal, Gunnar Sundstøl Eriksen

**Affiliations:** 1 Norwegian Institute of Bioeconomy Research, P.O. Box 115, N-1431 Ås, Norway; guro.brodal@nibio.no; 2 Norwegian Scientific Committee for Food Safety, P.O. Box 4404 Nydalen, N-0403 Oslo, Norway; inger.therese.lillegaard@vkm.no (I.T.L.); christiane.faste@vetinst.no (C.K.F.); annelise.brantsaeter@fhi.no (A.-L.B.); gunnar.eriksen@vetinst.no (G.S.E.); 3 Norwegian Veterinary Institute, P.O. Box 750 Sentrum, N-0106 Oslo, Norway; 4 Norwegian Institute of Public Health, P.O. Box 4404 Nydalen, N-0403 Oslo, Norway

**Keywords:** deoxynivalenol, DON, risk assessment, chronic exposure, *Fusarium graminearum*

## Abstract

Deoxynivalenol (DON) is the most common mycotoxin in Norwegian cereals, and DON is detected in most samples of crude cereal grain and cereal food commodities such as flour, bran, and oat flakes. The Norwegian Scientific Committee for Food Safety assessed the risk for adverse effects of deoxynivalenol (DON) in different age groups of the domestic population. This review presents the main results from the risk assessment, supplemented with some recently published data. Impairment of the immune system together with reduced feed intake and weight gain are the critical effects of DON in experimental animals on which the current tolerable daily intake was established. Based on food consumption and occurrence data, the mean exposure to DON in years with low and high levels of DON in the flour, respectively, were in the range of or up to two times the Tolerable Daily Intake (TDI) in 1-year-old infants and 2-year-old children. In years with high mean DON concentration, the high (95th-percentile) exposure exceeded the TDI by up to 3.5 times in 1-, 2- , 4-, and 9-year-old children. The assessment concluded that exceeding the TDI in infants and children is of concern. The estimated dietary DON intakes in adolescent and adult populations are in the range of the TDI or below, and are not a health concern. Acute human exposure to DON is not of concern in any age group.

## 1. Introduction

In Norway, cereals are produced in a cool, continental climate between 59° and 65° north. Barley cultivation amounts to about half the cereal acreage, while the rest is mainly wheat and oats. Most cereals are grown in monoculture. Domestic wheat production supplies 50%–70% of the human consumption, while barley and oats provide carbohydrates for the production of animal feed concentrate. This review is based on a risk assessment from the Norwegian Scientific Committee for Food Safety (VKM) [1] and is supplemented with some recently published results. The aim of the risk assessment was to provide a scientific evaluation for risk management related to the need of further measures to reduce the risks posed by exposure to mycotoxin-producing fungi in cereals.

Deoxynivalenol (DON) and analogues are the most common mycotoxins in Norwegian-grown cereals [2]. Previously, *Fusarium culmorum* was considered the main producer of DON in temperate regions of Europe, including Norway [2]. Towards the end of the 20th century, *Fusarium graminearum* became the main cause of DON contamination in European cereals [3]. A similar shift in *Fusarium* species towards increased occurrence of *F. graminearum* and reduction in *F. culmorum* prevalence has been reported from Norway [4]. When Hofgaard et al. [5] analysed 500 samples of spring wheat and oats from Norwegian farms collected during 2004–2009, DON concentrations were above 100 µg/kg in 66% of the oats and 70% of the spring wheat samples. The surveillance of mycotoxins in Norwegian cereal grains reported annual mean DON concentrations in oats from 100–2400 g/kg during the years 2002–2015, with the peak concentration in 2012 [6]. Analyses of domestic cereals harvested in 2011 revealed median DON levels of 150 µg/kg in barley, 383 µg/kg in wheat, and 1070 µg/kg in oats, while the maximum DON concentrations were five to seven times higher, and 7230 µg/kg was found in one oat sample [7]. In domestic samples collected during 2004–2009, *F. graminearum* DNA (≥0.1 pg per ng plant DNA) was detected in 69% of the oat samples and 62% of the spring wheat samples. There was a significant positive association between *F. graminearum* DNA content and DON+3-acetyl DON (3ADON), while *F. culmorum* DNA was not correlated with the DON content [5].

Although the 15-acetyl DON (15ADON) chemotype has been detected in Norway, the 3ADON chemotype is the most prevalent DON producer in the country [8]. Yli-Mattila et al. [9] found that 3ADON is dominant in Northern Europe, while in Central and South Europe, 15ADON has become the dominant chemotype. The Joint Food and Agriculture Organization of the United Nations/World Health Organization (FAO/WHO) Expert Committee on Food Additives (JECFA) concluded that the toxicity of the acetylated derivatives is equal to that of DON [10]. Global warming may in the future lead to higher precipitation in the main cereal-producing countries of North-Western Europe, which is predicted to increase the DON contamination of cereals by up to three times [11].

DON is reported to inhibit protein synthesis and induce the transcription of specific transcription factors related to inflammatory responses at low concentrations, while immunosuppression and cytotoxicity dominate at higher concentrations [12,13]. The main clinical effects of exposure to DON are reduced weight gain, inflammation at low doses, and reduced immune responses at higher doses. DON is shown to upregulate the expression of proinflammatory genes and several other genes related to (amongst others) communications between the innate and the adaptive immune systems and to cell–cell signaling [14]. DON also altered the expression of several genes involved in gastrointestinal disease, inflammatory disease, and response network. Furthermore, DON affected the gastrointestinal barrier, and induced a significant increase in levels of mRNA coding for interleukin (IL)-8, IL-1α, and IL-1β, tumor necrosis factor alfa (TNF-α) in porcine gut epithelial cells and in porcine jejunal explants alterations, which could be associated with intestinal inflammatory disease in humans [15]. DON increased the permeability through the gut epithelial layer both in vivo and in vitro [16]. Several national and international human risk assessments of DON have been published [10,17,18,19]. The current human tolerable daily intake (TDI) applied by both JECFA and the European Union is 1 µg/kg bw/day [18,19]. The TDI is based on the reduction in feed intake and weight gain in a two-year study in mice [20]. This TDI is also applied in Norway.

## 2. Human Exposure

Since the DON-producing *Fusarium* fungi grow and produce toxins in cereal grains, the exposure estimations are based on the occurrence in cereal-based products only. The occurrence data have been retrieved from the monitoring programme for mycotoxins in food for the Norwegian Food Safety Authority (NFSA) for the years 2008–2011 [1]. All samples were analysed by the Norwegian Veterinary Institute, which is the National Reference Laboratory for mycotoxins in food and feed. NFSA monitors DON levels in flour and not-finalised cereal products with the aim of limiting the required number of food categories to be sampled and rather increase the sample numbers in each category of flour instead. The decision was based on a desire to design a cost-effective monitoring programme.

The mycotoxin concentrations used in the exposure calculations are presented in Table 1. Mycotoxins were measured in four different flour products and in infant porridge. The highest and lowest mycotoxin concentrations during the years 2008–2011 for each flour type are presented in the table to illustrate the annual variations. Infant porridge was only analysed in 2008. The levels in samples below the limit of detection (LOD) were set to 0.5 × LOD when calculating the mean concentrations. The number of samples below the LOD were, however, low (<10% of the samples in each flour category), so this had little impact on the total mean concentrations of DON.

## 3. Cereal Consumption

Consumption data from three Norwegian dietary surveys were used. For 1-year-old [21] and 2-year-old [22] children, mothers filled in a semi-quantitative food frequency questionnaire covering the last 14 days. The study was conducted in 2006 and 2007. For 4-, 9-, and 13-year-old participants, food intakes were recorded by filling in a pre-coded food diary for four days [23,24]. The study was conducted in 2000 and 2001. For adults aged 18–70 years, two times 24-h recalls were chosen as the dietary assessment method. The study was conducted in 2010–2011 [25]. The consumption of different flour categories was extracted from these data based on calculations using standard recipes for all cereal-contaminated food items. A brief description of all dietary surveys used in the calculations is given in [1].

## 4. Estimations of Chronic DON Exposure in Humans

Occurrence data (Table 1) and consumption data (Table 2) were used to calculate the DON exposure for different age groups (Table 3). To illustrate the annual variation, the exposure was calculated using the mean concentrations from the year with the lowest DON concentrations in flour (mean low) and from the year with the highest DON concentrations in flour (mean high). The exposures were calculated using individual food consumption data, and the mean and high (95th percentile) intakes were calculated using the individual dietary intakes (Table 3).

DON was present in virtually all flour samples [1]. The current TDI is a group TDI applying to the sum of DON and its acetylated forms, but not DON-3 glucoside, which is probably also present in cereal samples. The acetylated forms and DON-3 glucoside were not included in the analysis of survey samples on which the present study is based, and the intake of these forms could therefore not be estimated. It has previously been shown that 3-ADON would add approximately 10% to the DON-levels, while 15-ADON was rarely present [26]. The occurrence of DON-3 glucoside in Norwegian grain has not been investigated in representative samples so far.

The estimated mean intakes of DON from cereal-based food were in the range of or exceeding the TDI for the 1- to 4-year-old children (Table 3). The estimated intake was highest for the 2-year-olds, probably due to high consumption of grain-based food in relation to a low body weight. In the year with the highest occurrence of DON in flour, the mean exposure was estimated to be twice as high as the TDI, and the high exposure was estimated to be 3.5 times higher than the TDI of 1 µg/kg bw in 2-year old children (Table 3). A considerably higher estimated exposure in children than in adults corresponded well with previous findings from a Norwegian study on the occurrence of DON in human urine, where DON concentrations were two-to-three-fold higher in the urine of 3–9 year-olds compared to adults [27]. In agreement with the exposure estimations in this paper, the biomonitoring study confirmed the ubiquitous exposure to DON in Norway. DON and DON-related metabolites were present in 99% of the Norwegian urine samples. The heterogeneous distribution of mycotoxins—including DON—in food makes sampling critical and calculations of the intake based on occurrence in food more uncertain. Use of biomonitoring methods to estimate the exposure circumvent these uncertainties [28]. Furthermore, the use of biomonitoring includes exposure from all sources, not only selected food items. The total exposure to DON was, however, not estimated by Brera et al. [27]. Furthermore, the total urine volume excretion was not recorded, and only samples of morning urine were analysed. The total exposure can therefore not easily be calculated from the data.

The estimated exposure of adults in Norway is higher than the exposures estimated for adult Swedish [29], Belgian [30], Dutch [31], French [32], and Spanish [33] populations. The estimated dietary intake of DON in Norwegian children is also higher than the corresponding estimated intakes in other European countries [29,30,31]. Since the Norwegian study is the only study in which the estimates are based only on the DON occurrence in flour and not food items as eaten, the discrepancies in the methodologies may be a contributing factor to these apparent differences. Furthermore, there are uncertainties related to both the methodologies used for the dietary surveys and for the representativeness of samples used in the calculations. However, to reduce the uncertainties related to sampling, the sampling in this study has been performed at the end of the processing of flour, and each sample is collected to represent one day’s production. A more detailed discussion of these uncertainties is given in [1].

It is of concern that the estimated exposure in young children exceeds the TDI. It should, however, be pointed out that the maximal estimated DON exposure in children (high consumers, maximal measured DON concentration) exceeds the TDI by not more than 3.5-fold, meaning a reduction of the safety margin established by JECFA by the same factor. JECFA derived the TDI by introducing a safety margin of 100 to the No Observed Adverse Effect Level (NOAEL) in a study on clinical effects in mice [18]. There is, however, an uncertainty in the exposure estimates, since known modified forms of DON (such as the acetylated forms and DON-3-glucoside) are not included in the exposure estimates. The toxicological relevance of the latter is also uncertain.

## 5. Estimations of Human Acute Exposure to DON 

An Acute Reference Dose (ARfD) of 8 µg/kg bw has been established for DON [10]. Oat flakes may have high DON concentrations (Table 1). Even if oat flakes are not the main contributor to the mean DON exposure, it is common to eat large portions of oat flakes as breakfast cereal or oatmeal porridge.

To illustrate a worst-case acute exposure, the amount of oat flakes or wheat bread a person would have to consume to reach the ARfD was estimated using the highest measured concentrations. A a 2-year-old child with a body weight of 12.8 kg, would have to consume 132 g oat flakes, corresponding to about 1100 g ready-to-eat oat meal porridge, or 91 g wheat, corresponding to 132 g based bread (approximately 3.5 slices of bread), to exceed the ARfD.

An adult with a body weight of 77.5 kg would have to consume 799 g oat flakes, corresponding to about 6.5 kg ready-to-eat oat meal porridge, or 552 g wheat, corresponding to 800 g bread (20 slices of bread), to exceed the ARfD.

The Norwegian Scientific Committee on Food Safety concluded that there is no concern for acute effects of DON from consumption of grains or grain-products in Norway [1].

## 6. Conclusions

DON is the most common mycotoxin in Norwegian cereals and it is the main mycotoxin of concern in domestic food products. Analyses of domestic grain show an increase in the mean concentrations of DON in barley, oats, and wheat during the last decade. Since the turn of the century, the most important DON producer—*F. graminearum*—has been detected at higher levels than previously in Norway. Of the *Fusarium* species infecting Norwegian cereals only *F. graminearum* is significantly and positively correlated to DON content. In the last decade, a precipitation increase during the cereal flowering season has been correlated with an increase in *Fusarium* infection rate and the occurrence of DON in oats and wheat.

Predicted future climate changes will lead to increased temperature and precipitation during the cereal flowering period in Northern Europe. This may increase the *Fusarium* infection rate and the occurrence of mycotoxins in cereals in the years to come.

The estimated mean exposures to DON in years with low and mean concentrations of DON in flour and oats, respectively, were in the range of or exceeded the TDI by almost two times in 1-year-old infants and 2-year-old children. In years with high mean DON concentrations, the high (95-percentile) exposures exceeded the TDI by up to 3.5 times in 1-, 2-, 4-, and 9-year-olds. The Norwegian Scientific Committee on Food Safety concluded that exceeding the TDI in infants and children is of concern, although the TDI is not a threshold for toxicity. The estimated dietary intakes of DON in adolescent and adult populations were equal to or below the TDI, and are therefore not a health concern. Acute exposure to DON is of no concern in any age group.

There are uncertainties related to the representativeness of the data. Each food sample was taken during one day of production and is considered to represent the production at the mill that day. There are uncertainties related to the analytical measurements. The laboratory estimated the expanded uncertainty to 40%. The uncertainty will, however, be to either side of the true value. The uncertainty is therefore likely to decrease with increasing number of samples. The human exposure estimates are based on the DON level in flour. There may be a certain reduction during food processing, but these changes are considered to be small due to the high stability of DON.

The occurrence data for DON in Norwegian cereals are scarce. There is a need for more systematic surveillance, especially focusing on products with high wheat and oat content. Grain infected by *Fusarium*-species in the field is more toxic than grain with the corresponding amount of pure toxin added. There is a need to identify the unknown factors in naturally-infected grain that add to the toxic effect.

## Figures and Tables

**Table 1:** Lowest and highest annual mean concentrations of deoxynivalenol (DON) in the years 2008–2011 in four flour categories and infant porridge included in the exposure calculations for the Norwegian population. All data are from the annual surveillance program for mycotoxins in food organized by the Norwegian Food Safety Authority, and are summarized in [1]. Copyright from Norwegian Scientific Committee for Food, 2013.

Flour Category	Lowest Mean Concentration ^a^			Highest Mean Concentration ^b^		
	Year ^a^	*n*	(µg/kg)	Year ^b^	*n*	(µg/kg)
Sieved wheat flour	2010	49	140	2008	65	210
Milled wheat flour	2010	42	121	2011	42	240
Wheat bran	2009	20	141	2008	23	383
Oat flakes	2011	30	165	2009	34	327
Infant porridge ^c^	2008	21	34	2008	21	34

^a^ The lowest mean concentration of four years (2008–2011). ^b^ The highest mean concentration of four years (2008–2011). ^c^ Infant porridge was only measured in 2008. The highest DON-concentration was found in oats-based infant porridge, which was used in the exposure estimations. Limit of detection: DON: 5–20 µg/kg.

**Table 2:** Consumption of different flour categories and infant porridge in different Norwegian age groups [21,22,23,24,25].

Age Group	Flour Category	Mean (g/day)	95-Percentile (g/day)
1-year-olds *n* = 1635	Sieved wheat flour	23	55
	Milled wheat flour	24	75
	Infant porridge	58	149
	Oat flakes	3	17
2-year-olds *n* = 1674	Sieved wheat flour	51	83
	Milled wheat flour	48	110
	Infant porridge	5	39
	Oat flake	8	32
4-year-olds *n* = 391	Sieved wheat flour	69	115
	Milled wheat flour	16	33
	Wheat bran	0	1
	Oat flakes	7	29
9-year-olds *n* = 810	Sieved wheat flour	98	167
	Milled wheat flour	20	44
	Wheat bran	0	1
	Oat flakes	9	46
13-year-olds *n* = 1005	Sieved wheat flour	109	204
	Milled wheat flour	18	45
	Wheat bran	0	1
	Oat flakes	5	32
Adults *n* = 1787	Sieved wheat flour	94	208
	Milled wheat flour	47	117
	Wheat bran	1	4
	Oat flakes	9	45

**Table 3:** Estimated exposure to deoxynivalenol (DON) (µg/kg bw/day) in years with low and high mycotoxin concentrations in flour for different age groups in Norway. The estimations are based on occurrence data in Table 1 and food consumption data in Table 2.

Age Group	Mean Low ^1^ (95-perc.) ^2^ (µg/kg bw/day)	Mean High ^3^ (95-perc.) ^2^ (µg/kg bw/day)
1-year-olds (*n* = 1635)	0.89 (1.8)	1.4 (3.1)
2-year-olds (*n* = 1674)	1.1 (1.9)	2.0 (3.5)
4-year-olds (*n* = 391)	0.73 (1.2)	1.1 (2.0)
9-year-olds (*n* = 810)	0.56 (1.02)	0.90 (1.6)
13-year-olds (*n* = 1005)	0.38 (0.72)	0.60 (1.1)
Adults (*n* = 1787)	0.27 (0.55)	0.45 (0.93)

^1^ Mean exposure in years with the lowest mean DON concentrations. ^2^ High exposure (95th percentile). ^3^ Mean exposure in years with the highest mean DON concentrations.
